# The Diversity, Multiplicity of Infection and Population Structure of *P. falciparum* Parasites Circulating in Asymptomatic Carriers Living in High and Low Malaria Transmission Settings of Ghana

**DOI:** 10.3390/genes10060434

**Published:** 2019-06-07

**Authors:** Zakaria Abukari, Ruth Okonu, Samuel B. Nyarko, Aminata C. Lo, Cheikh C. Dieng, Samson P. Salifu, Ben A. Gyan, Eugenia Lo, Linda E. Amoah

**Affiliations:** 1 Department of Biochemistry and Biotechnology, Kwame Nkrumah University of Science and Technology, Kumasi, Ghana; Zakus35@gmail.com (Z.A.); sspas@hotmail.com (S.P.S.); 2 Immunology Department, Noguchi Memorial Institute for Medical Research, University of Ghana, Accra, Ghana; ruth4destiny@yahoo.com (R.O.); amlosn@yahoo.fr (A.C.L.); bgyan@noguchi.ug.edu.gh (B.A.G.); 3 School of Medical Sciences, University of Cape Coast, Cape Coast, Ghana; captainsbn@gmail.com; 4 Department of Parasitology, University Cheikh Anta Diop, Dakar, Senegal; 5 Department of Biological Sciences, University of North Carolina at Charlotte, NC 28223, USA; cdieng@uncc.edu (C.C.D.); eugenia.lo@uncc.edu (E.L.); 6 West Africa Center for Cell Biology of Infectious Pathogens, University of Ghana, Accra, Ghana

**Keywords:** MSP2 genotyping, microsatellites, parasite, genetic diversity, malaria, multiplicity of infection

## Abstract

Background: Diversity in *Plasmodium falciparum* poses a major threat to malaria control and elimination interventions. This study utilized 12 polymorphic microsatellite (MS) markers and the Msp2 marker to examine diversity, multiplicity of infection (MOI) as well as the population structure of parasites circulating in two sites separated by about 92 km and with varying malaria transmission intensities within the Greater Accra Region of Ghana. Methods: The diversity and MOI of *P. falciparum* parasites in 160 non-symptomatic volunteers living in Obom (high malaria transmission intensity) and Asutsuare (low malaria transmission intensity) aged between 8 and 60 years was determined using Msp2 genotyping and microsatellite analysis. Results: The prevalence of asymptomatic *P. falciparum* carriers as well as the parasite density of infections was significantly higher in Obom than in Asutsuare. Samples from Asutsuare and Obom were 100% and 65% clonal, respectively, by Msp2 genotyping but decreased to 50% and 5%, respectively, when determined by MS analysis. The genetic composition of parasites from Obom and Asutsuare were highly distinct, with parasites from Obom being more diverse than those from Asutsuare. Conclusion: *Plasmodium falciparum* parasites circulating in Obom are genetically more diverse and distinct from those circulating in Asutsuare. The MOI in samples from both Obom and Asutsuare increased when assessed by MS analysis relative to MSP2 genotyping. The TA40 and TA87 loci are useful markers for estimating MOI in high and low parasite prevalence settings.

## 1. Introduction

Malaria is still highly endemic throughout Ghana, with the peak transmission season in most parts of the country coinciding with the major raining season. An increase in the implementation of malaria intervention programs, such as nationwide coverage of Indoor Residual Spraying (IRS), distribution and promotion of the use of Insecticide Treated Nets (ITNs) and larviciding, has resulted in a decline in the national prevalence of malaria from 50% in 2012 to 24.4% in 2016 [1,2]. The National Malaria Control Program (NMCP), in collaboration with the Ghana Health Service (GHS), has identified and prioritized some key measures, including the strict adherence to test (T), treat (T) and track (T) malaria, in addition to the use of Sulphadoxine-Pyrimethamine (SP) during pregnancy through the intermittent preventive treatment in pregnancy (IPTp) to sustain the continuous decline of malaria in the country [3] 

The main setbacks to the control and eventual elimination of malaria globally include the prevalence of asymptomatic *P. falciparum* carriers, the presence and distribution of highly diverse *P. falciparum* isolates (including drug resistant parasites) as well as infections harboring multiple *P. falciparum* isolates [4,5,6,7,8,9]. Asymptomatic individuals serve as transmission reservoirs, as these individuals do not treat and clear their infection but rather remain infectious for the duration of parasite carriage [6,10]. Genetic diversity in the malaria parasite is known to result from the recombination of distinct parasite clones during zygote formation within the mosquito mid gut [11,12]. This diversity has the potential to alter the conformation of antimalarial drug targets and render the parasites drug resistant [13] which will hinder malaria treatment outcome [14,15] as well as reduce the efficacy of a malaria vaccine [16,17]. Highly endemic malaria settings are prone to infections containing multiple *P. falciparum* isolates, primarily due to repeated exposure to mosquitoes infected with multiple parasite isolates [18]. Genetically diverse parasites can result in persistent infections, as some parasite isolates maybe resistant to the drug used for treatment and thus remain after the treatment [19] as well as enhance gametocyte production due to intra-host competition [20,21]. Parasite population structure, which provides the spatial distribution of the variant parasites, can be analyzed using similar tools used for parasite diversity measurements such as microsatellite analysis [22,23,24]. Allele frequencies and heterozygosity are key determinants used in measuring population structure [24].

Genetic diversity and multiplicity of *P. falciparum* infections are essential parasite indices that could determine the impact of malaria intervention programs as well as the endemicity of parasite infections in varying transmission settings [25,26,27]. The main tools use to estimate parasite diversity include PCR-based restriction fragment length polymorphism (PCR-RFLP) [28] single nucleotide polymorphism (SNP) [29] molecular typing of the polymorphic regions of merozoite surface protein (Msp)1 and Msp2, the glutamate rich protein (Glurp) genes followed by agarose gel electrophoresis [30] and analysis of unlinked microsatellite markers followed by capillary electrophoresis [31]. The main challenge with the most commonly used Msp1, Msp2 and Glurp antigenic markers for estimating parasite diversity is that these genes are under strong immune selection [32]. Selection pressure could bias diversity measurements with these markers, especially in endemic areas where individuals could harbor several parasite clones [33]. Finally, agarose gel electrophoresis is unable to effectively differentiate between closely sized fragments due to its poor discrimination [34].

## 2. Materials and Methods

### 2.1. Ethical Clearance

The Institutional Review Board (IRB) of the Noguchi Memorial Institute for Medical Research (NMIMR) gave approval for the original study (NMIMR-IRB CPN 089/14-15). Consent for the reuse of the samples was incorporated into the original consent form. Participants were enrolled only after written informed consent was obtained. Written parental consent was obtained from the parents or guardians of participants who were minors.

### 2.2. Study Site and Sample Collection

The original cross sectional survey recruited 160 non-febrile adults and children aged between 8 and 60 from Obom and Asutsuare, within the Greater Accra Region of Ghana. Asutsuare and Obom are only about 92 km apart. Obom is a high malaria transmission area with parasite prevalence estimated by microscopy to be 35% [35] and Asutsuare is a low transmission area with microscopy estimated parasite prevalence set at 8.9% [36]. Venous whole blood (5 mL) from each participant was collected into EDTA vacutainer tubes. A small drop of blood was used to prepare thick (6 µL) and thin (2 µL) blood films, after which the blood was separated into plasma and packed blood cell pellets. Both the plasma and packed blood cell pellets were stored at −20 °C. This study randomly selected 80 packed blood cell samples from the set of samples stored from each site.

Thick and thin blood films were prepared and processed according to the WHO protocol for blood smear preparation and subsequent Giemsa staining [37]. One microscopist examined both the thick and thin films and a second microscopist performed quality control on 10% of the slides.

### 2.3. DNA Extraction

Genomic DNA (gDNA) was extracted from the frozen blood cell pellets using the zymo Quick gDNA Blood Mini prep extraction kit following the manufacturer’s protocol (Zymo Research, Irvine, CA, USA). Approximately 100 µL of pelleted blood cells was used for each sample DNA extraction. The concentration of each extracted gDNA was determined using a Nanodrop 2000C (Thermo Fisher Scientific, Waltham, MA, USA).

### 2.4. Msp2 Genotyping

The polymorphic region of Msp2 block 3 was genotyped using nested PCR [38,39] Briefly, a 15 µL primary reaction was performed containing 100 nM of the Msp2 forward (M2-OF) and reverse (M2-OR) primers, 200 nM of dNTP, 2 mM of MgCl_2_, 0.5 U of OneTaq DNA Polymerase (New England Biolabs, Ipswich MA, USA) and 3–5 ng/µL of gDNA. The nested reaction contained 200 nM of dNTP, 1.8 mM of MgCl_2_, 200 nM each of the N5 rev/S1w-f and M5 rev/S1w-f primer sets, 0.5 U of OneTaq DNA Polymerase and 3 µL of the primary reaction products. Genomic DNA from KI (MRA-155G) and 3D7 (MRA-102G) were used as positive controls, and water was used as the negative control (no template control). Amplifications were performed using a BiometraT Advance thermocycler (Gottingen, Germany). The PCR products as well as a 50 bp ladder (Thermo Fisher Scientific, Waltham, MA, USA) were resolved on a 2% agarose gel and visualized using UV illumination.

### 2.5. Microsatellite Analysis

Twelve distinct polymorphic microsatellites markers were used to genotype the samples using a hemi-nested PCR protocol described by Anderson et al., (1999) [31] with slight modification. The markers included Poly α, TA40, ARA2, TA87, TAA81, TAI, TA42, TA60, 2490, PfG377, TA109 and PfPK2. Briefly, the primary reaction was carried out in a solution of 15 µL total containing 200 nM dNTP, 3 mM MgCl_2_, 0.1 µM each of the unlabeled forward and reverse primer pairs, 0.05 U One Taq DNA Polymerase (New England Biolab, Ipswich, MA, USA) and about 30–50 ng of gDNA template. The secondary reaction contained the same reagents as the primary reaction with the exception of the primer pair that included 0.3 µM of a fluorescently labeled forward primer (6-FAM, HEX and Atto 565) and 0.1 µM of the unlabeled reverse primer used in the primary reaction (Table S1 in Supplementary Materials) with 3 µL of the primary reaction product in a final volume of 15 µL [31]. The fluorescently-labeled PCR products were separated using an Applied Biosystems 3130 series Genetic Analyzer and visualized using the GeneMapper software v 5.0 (Applied Biosystems, Foster City, CA, USA).

### 2.6. Data Analyses

The multiplicity of a *P. falciparum* in an individual sample is defined as the number of distinct parasite clones identified in a specific DNA sample. For this study, a sample was considered as a monoclonal infection when only one amplified product was detected for each of the gene loci. Any sample that produced more than one amplified product at any of the gene loci was considered as a polyclonal infection [27] The sizes of all the amplicons obtained after Msp2 PCR were estimated relative to the molecular weight marker. The average MOI of all samples collected from a given site was determined as the ratio of the total number of distinct parasites clones (distinct fragments) obtained for a specific marker relative to the number of samples that tested positive for that marker at each site. Descriptive column statistics, geometric mean (used to report mean MOI and PD) and unpaired T test (Mann and Whitney test) in GraphPad prism version 5.0 was used to analyzed Msp2 data. The frequency of distribution of the 3D7 and FC27 family species at each site were determined as the ratio of the number of PCR products to that of the total number of Msp2 products amplified [38,39,40]. *p* Value ≤ 0.05 was considered statistically significant.

Convert 1.31 was used to convert allelic excel report file into a notepad (text editable) file. The gene analysis software, GenAIEx 6.5 [41] and Arlequin ver 3.0 [42], were used for the population genetic analysis. The microsatellite data were analyzed in two ways for diversity measures. The first analysis included only monoclonal and biclonal samples. Samples were considered as bi-clonal when two equally dominant alleles were detected in a single locus (Table S3). We separated the genotypes of the two strains and included them in the analyses. For samples that showed >1 allele in two or more loci, we were unable to unambiguously differentiate the genotypes of the different strains. Thus, these samples were discarded in the analyses. The second analysis involved transformation of genotype data into a binary format that showed presence or absence of a specific allele for each of the samples (Table S4). All samples were included to infer overall genetic diversity in the parasite population, but this analysis did not encounter the genotype difference for each of the parasite strains. For both datasets, the number of effective alleles and expected heterozygosity were estimated for each study population in GenoDive v2.0b27 [43]. Heterozygosity (*He*) was determined using the formula *He* = (n/n – 1) (1 − ∑p_i_^2^), where n is the number of *P. falciparum* samples and p_i_ is the frequency of alleles in each of the SSR locus. The *He* ranged from zero (no genetic diversity in alleles) to 1 (high allelic diversity in samples) [27,31].

With the genotypic data, we further calculated pairwise squared Euclidean distances based on the number of times a certain allele was found in the two parasite strains. Neighboring-joining tree was constructed using T-REX [44] to show the genetic relatedness among the parasites. In addition, a model-based Bayesian method implemented in STRUCTURE v2.3.4 was performed to examine partitioning of individuals to genetic clusters [45]. The posterior probability of each value was used to detect the modal value of Δ*K*, a quantity related to the second order rate of change with respect to *K* of the likelihood function [46]. Posterior probability values were estimated using a Markov Chain Monte Carlo (MCMC) method. A burn-in period of 500,000 iterations followed by 10^6^ iterations of each chain was performed to ensure convergence of the MCMC. Each MCMC chain for each value of *K* was run ten times with the ‘independent allele frequency’ option that allows individuals with ancestries in more than one group to be assigned into one cluster. Individuals were assigned into *K* clusters according to membership coefficient values (Q) ranged from 0 (lowest affinity to a cluster) to 1 (highest affinity to a cluster). The partitioning of clusters was visualized with DISTRUCT [47].

## 3. Results

### 3.1. Demographics of Study Participants

In Table 1, the prevalence of asymptomatic *P. falciparum* carriers identified by microscopic evaluations of thick and thin blood films was estimated at 33.75% (27/80) in Obom and 3.75% (3/80) in Asutsuare. No significant differences were observed in ages and gender of the volunteers from both study sites (T test, *p* > 0.05). The prevalence of asymptomatic parasite carriers by microscopy in Obom was significantly (two tailed T test, *p* < 0.001) higher than in Asutsuare.

### 3.2. Parasite Diversity and Multiplicity of Infection (MOI)

#### 3.2.1. Msp2 Estimates of MOI

**Figure 1:**
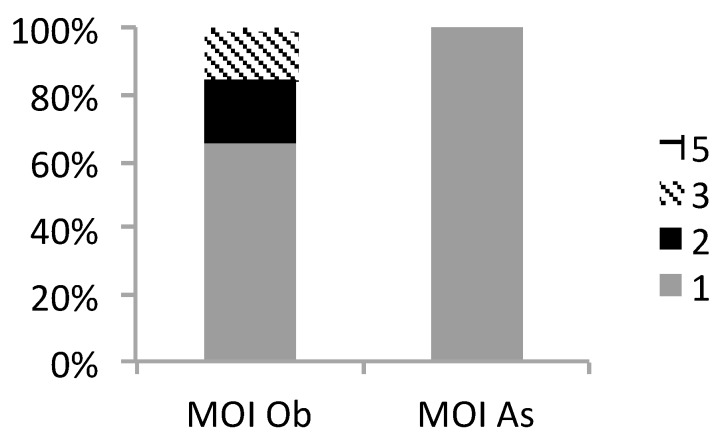
Multiplicity of *P. falciparum* infection determined by Msp2. A graphical representation of the total number of fragments (gray (**1**), black (**2**), diagonal stripes (**3**) and checkered (**5**)) detected in each sample expressed as a % of the number of samples positive for Msp2 at Obom (Ob) and Asutsuare (As).

In Table 2 and Figure 1, all the asymptomatic *P. falciparum* infections identified in Asutsuare were clonal at the Msp2 locus with a MOI of 1, whilst in Obom the MOI ranged from 1 to 5, with a geometric mean MOI (95% CI) of 1.366 (1.215–1.536). Multiple parasite clones were detected in 35% (21/60) of the samples in Obom, with 71% (15/21) consisting of infections containing both the 3D7 and the FC27 allelic families. Four samples belonging exclusively to the FC27 family were clonal, as opposed to those exclusively in the 3D7 family where six of the samples were multiclonal. Although parasites with the 3D7 allele were three times more prevalent than the FC27 allele in Obom, the difference was not statistically significant (Mann–Whitney T test; *p* = 0.7459, Mann–Whitney U = 486.0). There were too few Msp2 positive samples in Asutsuare to do statistical analysis and so the trends observed in Asutsuare will be discussed in general terms.

#### 3.2.2. Msp2 Estimates of Genetic Diversity

**Figure 2:**
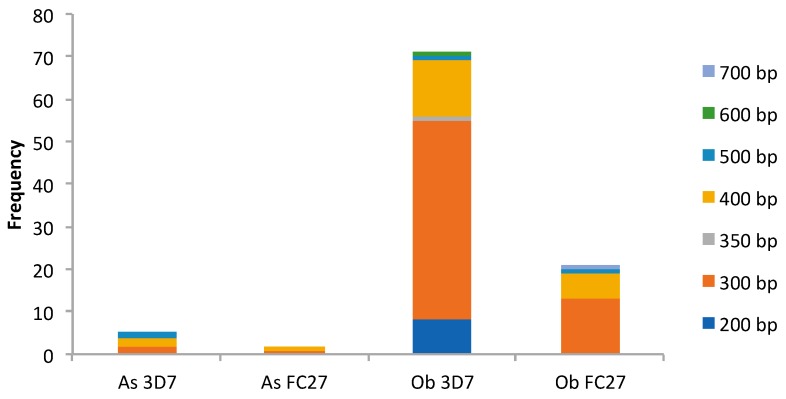
Msp2 gene diversity. As, Asutsuare; Ob, Obom; FC27 and 3D7 are the two Msp2 allelic families. Each color (dark blue (200 bp), orange (300 bp), gray (350 bp), honey (400 bp), light blue (500 bp), green (600 bp) and periwinkle (700 bp)) represents a fragment size (bp), irrespective of the allelic group.

In Table 2 and Figure 2, Msp2 genotyping on the samples collected from Obom identified 56 samples as positive for the 3D7 allelic family, with fragment sizes ranging from 200 bp to 600 bp, and 20 samples were positive for the FC27 allelic family, with fragment sizes ranging from 300 bp to 700 bp. There were 16 samples that were positive for both alleles. In Asutsuare, five samples belonged to the 3D7 allelic family and two samples belonged to the FC27 allelic family. The fragment sizes range between 300 bp to 500 bp and 300 bp to 400 bp (rounded to the nearest 50 bp) for the 3D7 and FC27 alleles family, respectively. No single infection in Asutsuare contained parasites belonging to both allelic families.

#### 3.2.3. MOI Determined by Microsatellite (MS) Analysis

**Figure 3:**
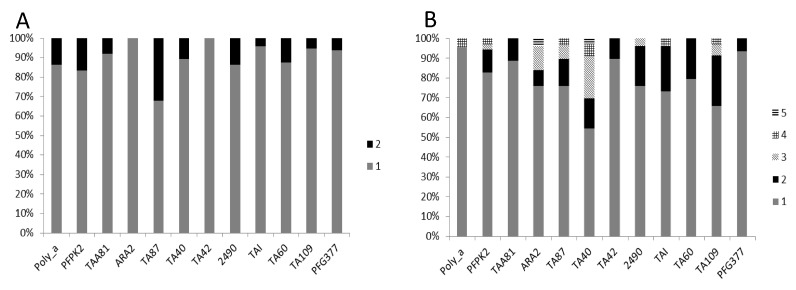
Distinct number of clones identified at each MS marker. (**A**), samples from Asutsuare and (**B**), samples from Obom. The color code representing the different number of clones for both graphs: gray (1), black (2), light gray polka dots (3), checkered (4) and black horizontal lines (5).

Samples were classified as either clonal (monoclonal), containing only one allele per marker, biclonal, containing a maximum of two alleles at a single marker or poly clonal, containing more than two alleles at one marker or multiple alleles at two or more markers. For the purpose of this study, a sample containing two alleles at two separate markers, which could be either biclonal or polyclonal, was considered as polyclonal. In Figure 3, samples from Obom contained polyclonal parasites with higher multiplicities of infection. Five alleles were found at the ARA2 and the TA40 loci in samples from Obom.

In Obom, the highest level of clonality (95.7%) was recorded at the Poly_α loci, where 22 out of the 23 samples contained a single parasite allele; whereas the lowest of 54.5% was recorded at the TA40 loci, where 18 out of the 33 samples contained a single parasite allele. In Asutsuare, the highest number of alleles at any one locus was two, with 68% (8/25) of the samples having two alleles at the TA87 loci and all (100%) the samples at the ARA2 and TA42 loci containing a single allele. Some of the samples (11 from Obom and 33 from Asutsuare) that were negative by Msp2 genotyping were randomly selected for microsatellite analysis. In Obom, 36.4% (4/11) of the Msp2 negative samples yielded amplified products after MS analysis, and 75% (3/4) of these samples were multiclonal. In Asutsuare, 54.5% (18/33) of the Msp2 negative samples tested positive by MS analysis. Amongst these samples, 39% (7/18) were monoclonal, with a single allele identified at all the 12 loci, or 50% (9/18) were biclonal, with 2 alleles identified at one of the 12 loci, and 11.1% (2/18) were polyclonal.

### 3.3. Genetic Diversity Estimated by Microsatellite Analysis

**Figure 4:**
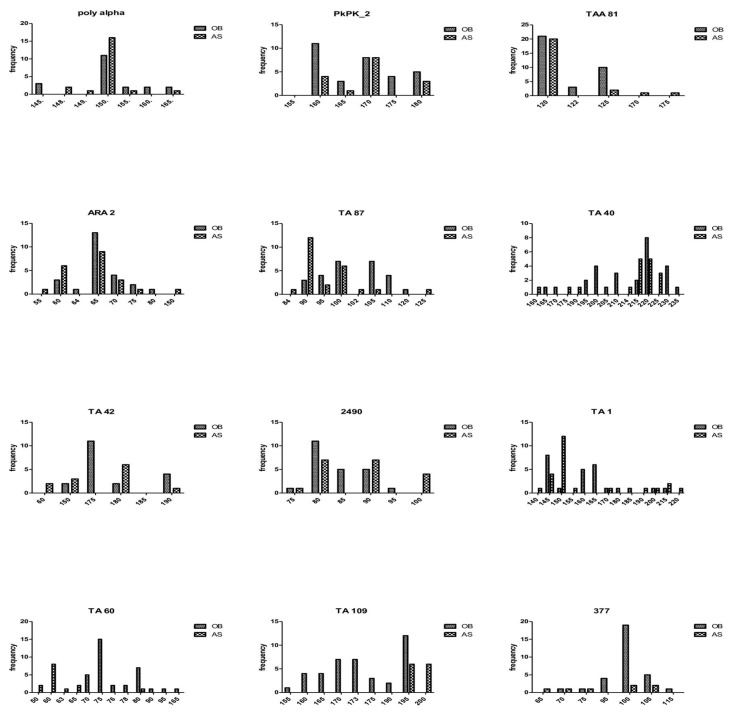
Allelic diversity at the 12 microsatellite markers. Alleles (vertical axis) were scored by GeneMapper v5 and GenAIEx and used to generate allelic frequencies (Obom in blue and Asutsuare in orange bars) in both populations.

In Figure 4 and in Table S2, the highest and lowest number of alleles at any one locus in samples from Obom was found at the TA40 (13) and TA42 (4) locus, respectively, and at the TA87 (10) and the TA109 (2) locus, respectively, in samples from Asutsuare.

**Figure 5:**
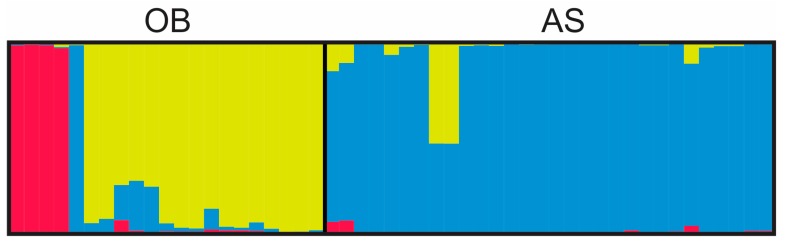
Bayesian barplot showing genotyping structure of *P. falciparum.* OB, Obom and AS, Asutsuare. Three genetic clusters were detected (color in pink, yellow and blue). Samples of OB had predominantly the yellow cluster followed by the pink and blue clusters; samples from AS had mostly the blue cluster. Only monoclonal and biclonal samples, of which multilocus genotypes were identified, were included in this analysis.

In Table 3, the expected heterozygosity showed no significant difference between samples from the two study sites (*p* > 0.05, by one-tailed T test). In Figure 5, the three most probable genetic clusters were detected among the *P. falciparum* samples. The genetic composition of samples between Obom and Asutsuare was distinct. The yellow cluster was more predominant in Obom relative to the pink and blue clusters among the samples. For Asutsuare, the blue cluster was predominant among all samples.

**Figure 6:**
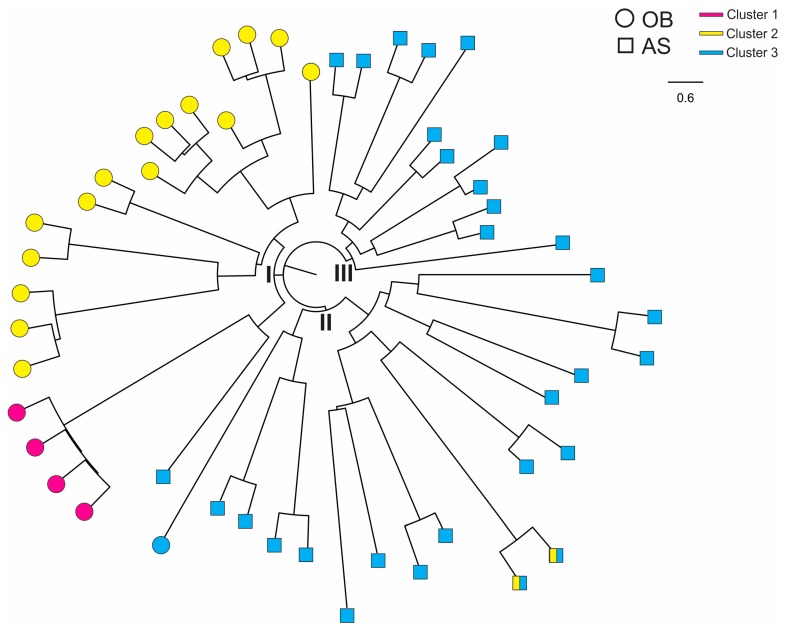
Neighbor-joining tree showing genetic relatedness among the *P. falciparum* isolates. OB, Obom (as circles) and AS, Asutsuare (as squares). Color (pink, yellow and blue) denoted the corresponding cluster for each sample (Figure 4). Samples from OB were found in clade I and samples from AS in clades II and III.

In Figure 6, samples from Obom had all the three genetic clusters and appeared to be more genetically diverse than those from Asutsuare. Based on the tree analysis, samples of the yellow and pink clusters were sister to one another in a single clade (I). Samples from Asutsuare were divided into two clades (II and III). While the Obom samples were genetically distinct from the Asutsuare ones, sample AS100 from Asutsuare was sister to the pink cluster that contained the Obom samples. Likewise, one sample from Obom was nested within the clade that contained the Asutsuare samples.

## 4. Discussion

Most parasite diversity studies conducted on malaria parasites in Ghana utilize *Plasmodium* merozoite surface 1 and/or 2 genotyping [19,38,39,48,49] with very few studies utilizing microsatellite analysis [50,51]. This study uses microsatellite analysis to confirm the diversity and complexity of *P. falciparum* parasite infections in southern Ghana, and is consistent with the Msp2 genotyping using amplification fragment length polymorphisms followed by agarose gel electrophoresis. Polyclonal infections were more prevalent in Obom than in Asutsuare, where no polyclonal infection was recorded at the Msp2 locus and the highest multiplicity recorded at any microsatellite marker was two. The enhanced immunity in people living in high transmission settings [52] could be the reason why one-third of the samples collected from asymptomatic *Plasmodium* carriers in Obom harbored multiple co-infecting variant parasites, whilst all the samples from Asutsuare were mostly clonal, likely due to reduced exposure and consequently lowered immunity. Despite the higher prevalence of polyclonal infections at the Msp2 locus in Obom, the geometric mean MOI at the Msp2 locus was relatively low and not significantly different from that reported in Asutsuare. The low report, especially for Obom could likely be due to the fact that the samples used for study were collected during the off-peak season as MOI has also been reported to be lower in samples collected during the off peak season relative to those collected in the peak season [53] Another reason for the low MOI recorded in Obom could be the inclusion of adults in the sampling, as adults have been suggested to harbor infections with lower MOI than children [54,55] This is contrary to an earlier report where MOI determined by Msp2 genotyping in asymptomatic children in Obom was significantly higher than MOI in children from Abura [19,39] a community with low parasite prevalence, similar to Asutsuare. Similarly, the mean MOI determined by MS analysis in this study was lower than that reported for a study that genotyped 10 polymorphic microsatellite markers on samples from symptomatic individuals collected between 2005–2009 from Gambia (2.1), Guinea (4.0), Guinea Bissau (2.6) and Senegal (2.2) [56] Differences in reported MOI could be due to the fact that symptomatic individuals have been suggested to harbor higher numbers of parasite clones [57,58,59,60].

The allelic range of both allelic families, 3D7 and FC27 of parasite, from Obom were wider (300 bp–600 bp) than the fragment range of parasites in Asutsuare (400 bp–500 bp), which is similar to a study in the Thai–Myanmar border, where the endemic site had parasites with higher allelic range compared to the low parasite prevalence site [61] An earlier study in Obom also identified the 3D7 family to have a higher allelic range than the 3D7 family of parasites from Abura, a low parasite prevalence setting, however the FC27 allelic family range was similar in Obom and Abura [38] The higher frequency of parasites belonging to the 3D7 family in both sites reinforces other studies, which found the 3D7 strain to be higher than FC27 strain [25,62].

The total number of *P. falciparum* alleles identified at each of the 12 microsatellite markers varied greatly at Obom compared with Asutsuare. Whilst the highest number of alleles identified at Asutsuare was two for a maximum of three loci in a single sample, up to five alleles were identified in samples from Obom, and multiple alleles were identified in up to five different markers. These results indicate a tremendous level of within-host diversity, which was not observed by Msp2 genotyping. This outcome could be as a result of the predicted endemicity of the parasite population in Obom relative to the limited transmission at Asutsuare [56] The genetic diversity determined by MS for both study sites was moderate in Asutsuare (*He*; 0.24–0.76) and high in Obom (*He*; 0.53–0.88). Similar heterozygosity has been reported in other malaria endemic countries where *He* was reported to be about 0.8 in Mali [26] 0.79 in Nigeria [22] 0.72 in Senegal, 0.78 in Guinea Bissau, 0.78 in Guinea and 0.75 in Gambia [56] Despite the difference in transmission intensity between Obom and Asutsuare, samples were clearly structured and differentiate from one another, suggestive of limited gene flow between the two sites.

The enhanced sensitivity and increased abundance of MS markers relative to agarose gel based Msp2 genotyping [34,63,64,65] was confirmed in this study, where some samples that did not yield any amplified product after Msp2 genotyping tested positive and produced amplified fragments after MS typing. It also suggests that MS typing is able to identify parasites in infections that are falsely classified as negative by Msp2 genotyping due to low parasite density. A previous study identified the efficiency of Msp2 genotyping to be under 90% [38] making room for false negative samples to be identified by other parasite identification techniques.

The complexities of the parasites in samples from Asutsuare, which were negative by Msp2 genotyping, were low (mostly clonal) and similar to the Msp2 positive samples. A similar occurrence was observed in Obom, where the complexity of the Msp2 negative samples was as high as the Msp2 positive samples. These findings suggests that parasite complexity is independent of parasite density, as has been previously reported [66] but it is more likely to be associated with transmission intensity and the seasons during which the samples were collected. This is not necessarily contrary to previous reports, which have found the complexity of parasites to be significantly related to parasites density in Ghana [19] and elsewhere [14,55,67] as we did find the diversity and complexity to be different between the two sites with known differences in parasite density.

## 5. Conclusions

Asymptomatic infections in southern Ghana are predominantly multiply infected and the *P. falciparum* parasites circulating in Obom are genetically distinct from those circulating in Asutsuare. The TA40 and TA87 loci are useful markers for estimating the multiplicity of infection in high and low parasite prevalence settings respectively.

## Figures and Tables

**Table 1:** Demographic features of study participants.

Parameter	Obom (80)	Asutsuare (80)	*p* Value
Age (yrs) Mean (SEM)	17.6 (1.1)	19.6 (1.2)	
Min–Max	6–45	3–66	
Males N (%)	41 (52%)	43 (54%)	
Microscopy N (%)	27 (33.75%)	3 (3.75)	0.001
Positive, N (%)	27 (33.75%)	3 (3.75)	<0.001
Min–Max	32–5080	16–400	
Geometric mean PD (95% Cl)	318.8 (180.6–562.5)	*	

N, count; yrs, years; PD, parasite density; * there were too few samples for the Geometric mean PD to be calculated in Asutsuare; Min, minimum; Max, maximum.

**Table 2:** Genetic diversity within Block 3 of Msp2.

	Marker	F (*n*)	Range (bp)	MOI (GM)
Obom (61)				
	3D7	68 (6)	200–600	1.37
	FC27	24 (4)	300–700	
Asutsuare (7)				
	3D7	5 (3)	300–500	1
	FC27	2 (2)	300–400	

N, total number of Msp2 positive samples; MOI, average multiplicity of infection; F, number of amplified fragments; n, number of variants; Range, minimum and maximum fragment sizes. The number of positive samples at each site is in brackets.

**Table 3:** Genetic diversity of the microsatellite markers.

Dataset	Study Site	Samples (*n*)	Effective Alleles (*n*)	Heterozygosity
Genotype				
	AS	21 *	2.961	0.67
	OB	30 *	3.384	0.694
Binary				
	OB	35	1.233	0.158
	AS	25	1.196	0.135

* Number of clones included in the analysis; *n*, number; AS, Asutsuare; OB, Obom. The data was analyzed as genotype as well as binary data. Only monoclonal and biclonal samples were included in the analysis for the genotype dataset and parasite clones with two equally dominant alleles at a single locus were separated. All samples were included in the binary analysis, and alleles were scored as present or absent.

## Supplementary Materials

The following are available online at https://www.mdpi.com/2073-4425/10/6/434/s1, Table S1: Primer sequences; Table S2: Characteristics of the 12 microsatellite markers; Table S3: Genotype data of 12 microsatellite loci for 51 monoclonal and biclonal samples; Table S4: Binary data of microsatellite alleles among all 60 *P. falciparum* infected samples from Obom (OB) and Asutsuare (AS). Each allele was coded as presence (1) or absence (0) for each sample. Monoclonal and polyclonal samples were included in the analyses.
